# Supplementary material for: Association between oral microbiome and five types of respiratory infections: a two-sample Mendelian randomization study in east Asian population
Source: Front Microbiol. 2024 Apr 10;15:1392473. doi: 10.3389/fmicb.2024.1392473 (PMC11039966; doi:10.3389/fmicb.2024.1392473)
Supplement: Supplementary file 1 [file Data_Sheet_1.zip › Supplementary Materials/MR plots of saliva/Chronic sinusitis/s__Gemella_haemolysans_B_mgs_2903/leave_one_out.pdf]

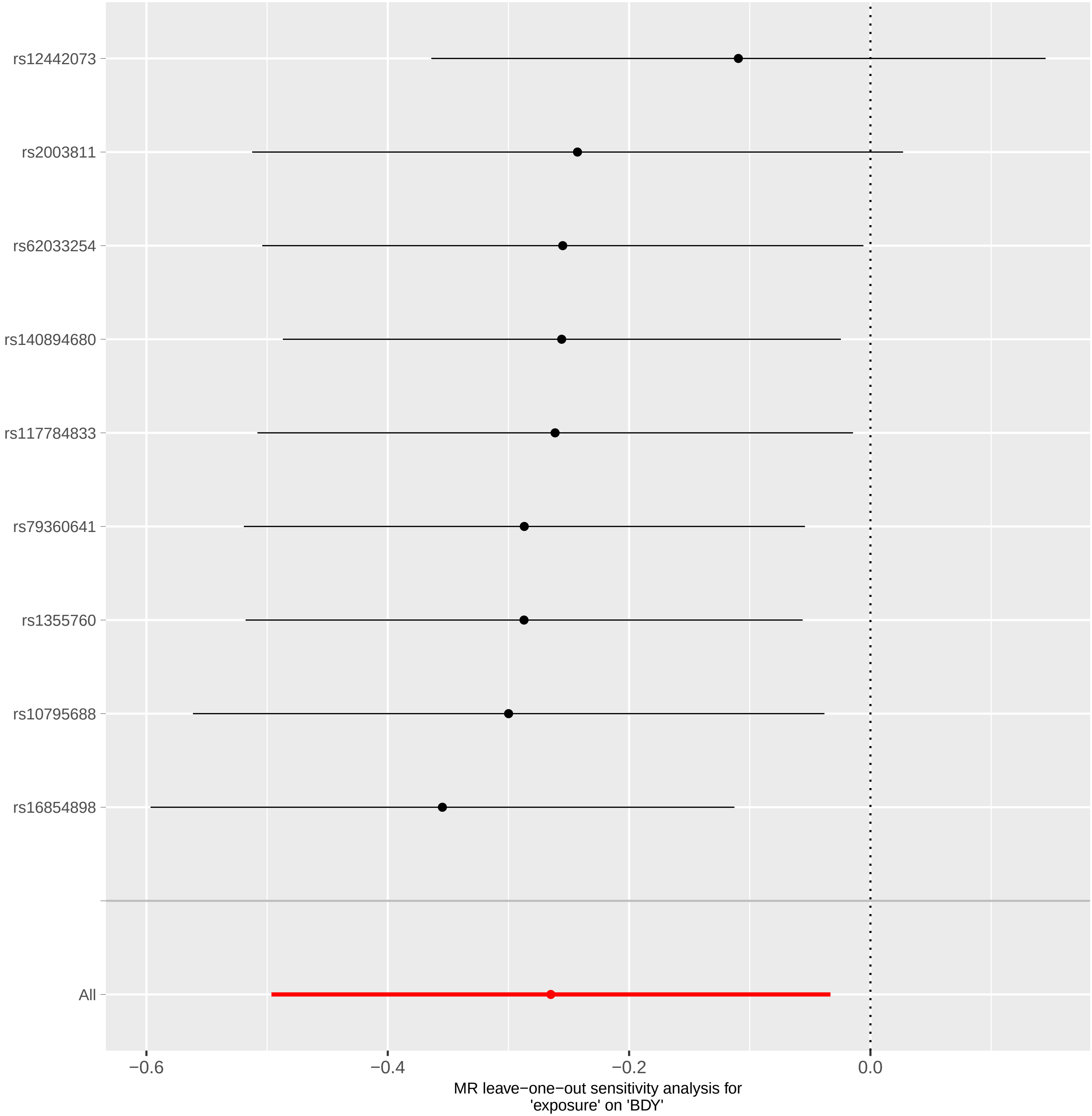
rs12442073
rs2003811
rs62033254
rs140894680
rs117784833
rs79360641
rs1355760
rs10795688
rs16854898
All
−0.6
−0.4
−0.2
0.0
MR leave−one−out sensitivity analysis for
'exposure' on 'BDY'
